# Blockade of Sialylation with Decrease in Polysialic Acid Levels Counteracts Transforming Growth Factor β1-Induced Skin Fibroblast-to-Myofibroblast Transition

**DOI:** 10.3390/cells13121067

**Published:** 2024-06-19

**Authors:** Bianca Saveria Fioretto, Irene Rosa, Alessia Tani, Elena Andreucci, Eloisa Romano, Eleonora Sgambati, Mirko Manetti

**Affiliations:** 1 Section of Anatomy and Histology, Department of Experimental and Clinical Medicine, University of Florence, Largo Brambilla 3, 50134 Florence, Italy; biancasaveria.fioretto@unifi.it (B.S.F.); irene.rosa@unifi.it (I.R.); alessia.tani@unifi.it (A.T.); 2 Imaging Platform, Department of Experimental and Clinical Medicine, University of Florence, Largo Brambilla 3, 50134 Florence, Italy; 3 Section of Experimental Pathology and Oncology, Department of Experimental and Clinical Biomedical Sciences “Mario Serio”, University of Florence, Viale Morgagni 50, 50134 Florence, Italy; e.andreucci@unifi.it; 4 Section of Internal Medicine, Department of Experimental and Clinical Medicine, University of Florence, Largo Brambilla 3, 50134 Florence, Italy; eloisa.romano@unifi.it; 5 Department of Biosciences and Territory, University of Molise, Contrada Fonte Lappone, Pesche, 86090 Isernia, Italy; eleonora.sgambati@unimol.it

**Keywords:** skin fibroblasts, myofibroblasts, skin fibrosis, fibroblast-to-myofibroblast transition, TGFβ1, sialylation, polysialic acid, sialyltransferase inhibitor

## Abstract

Aberrant sialylation with overexpression of the homopolymeric glycan polysialic acid (polySia) was recently reported in fibroblasts from fibrotic skin lesions. Yet, whether such a rise in polySia levels or sialylation in general may be functionally implicated in profibrotic activation of fibroblasts and their transition to myofibroblasts remains unknown. Therefore, we herein explored whether inhibition of sialylation could interfere with the process of skin fibroblast-to-myofibroblast transition induced by the master profibrotic mediator transforming growth factor β1 (TGFβ1). Adult human skin fibroblasts were pretreated with the competitive pan-sialyltransferase inhibitor 3-Fax-peracetyl-Neu5Ac (3-Fax) before stimulation with recombinant human TGFβ1, and then analyzed for polySia expression, cell viability, proliferation, migratory ability, and acquisition of myofibroblast-like morphofunctional features. Skin fibroblast stimulation with TGFβ1 resulted in overexpression of polySia, which was effectively blunted by 3-Fax pre-administration. Pretreatment with 3-Fax efficiently lessened TGFβ1-induced skin fibroblast proliferation, migration, changes in cell morphology, and phenotypic and functional differentiation into myofibroblasts, as testified by a significant reduction in *FAP*, *ACTA2*, *COL1A1*, *COL1A2*, and *FN1* gene expression, and α-smooth muscle actin, N-cadherin, COL1A1, and FN-EDA protein levels, as well as a reduced contractile capability. Moreover, skin fibroblasts pre-administered with 3-Fax displayed a significant decrease in Smad3-dependent canonical TGFβ1 signaling. Collectively, our in vitro findings demonstrate for the first time that aberrant sialylation with increased polySia levels has a functional role in skin fibroblast-to-myofibroblast transition and suggest that competitive sialyltransferase inhibition might offer new therapeutic opportunities against skin fibrosis.

## 1. Introduction

Skin fibrosis is a consequence of an exaggerated and prolonged wound healing response clinically manifesting as thickened, tightened, and hardened cutaneous areas that become firmly adherent to the underlying soft tissues and, ultimately, may cause considerable morbidity with loss of flexibility, joint contractures, and disfigurement [1,2]. The wide and heterogeneous spectrum of fibrotic skin conditions includes disorders as various as hypertrophic and keloid scars, morphea (localized scleroderma), systemic sclerosis (scleroderma), chronic graft-versus-host disease, nephrogenic fibrosing dermopathy, Dupuytren’s contracture, eosinophilic fasciitis, and chemical- and radiation-induced and post-burn trauma skin fibrosis [1,2,3,4,5,6,7,8,9,10]. These diseases are characterized by exceptionally high medical needs and, very frequently, poor treatment effects [11,12].

Despite different etiologies and disease-specific pathophysiologic processes, especially immune, autoimmune, and inflammatory mechanisms, that lead to cutaneous fibrosis, the abnormal and excessive accumulation of extracellular matrix (ECM) constituents in the skin is universally governed by proliferation, chronic activation, and transition of fibroblasts toward a myofibroblast phenotype [13,14,15]. Indeed, at variance with physiologic wound healing in which α-smooth muscle actin (α-SMA)-expressing myofibroblasts undergo apoptosis after wound contraction, the aberrant persistence of myofibroblasts results in the formation of a scar tissue consisting in a hyperplastic, disorganized, and stiff ECM particularly rich in collagens, hyaluronan, and fibronectin [11,13,14,15,16,17,18]. In such a scenario, ECM stiffness and myofibroblast-ECM interactions have emerged as prominent mechanical cues that drive skin fibrosis progression by perpetuating myofibroblast differentiation and evasion of apoptosis in a kind of vicious circle [13,18,19]. Infiltrating immune cells also play a key role not only in initiating but also in amplifying the fibrotic response through the secretion of several soluble mediators such as transforming growth factor β (TGFβ), accountable for fibroblast-to-myofibroblast transition, promotion of ECM synthesis and deposition, and further recruitment of inflammatory/immune cells [2,11,16,18,20,21].

Sialic acids, primarily found as terminal components of glycans in the structure of glycoproteins and glycolipids of the cellular glycocalyx, are important mediators of cell function, particularly with regard to cell signaling interactions with the surrounding microenvironment [22,23]. The most abundant glycocalyx sialic acid is N-acetylneuraminic acid (Neu5Ac), which often terminates the glycan structures and whose active form, i.e., cytidine monophosphate (CMP)-Neu5Ac, is used as donor substrate in enzymatic reactions catalyzed by sialyltransferases that transfer Neu5Ac to the growing glycan chains of glycoproteins and glycolipids [22,23]. Among glycans, polysialic acid (polySia) is a linear homopolymer of α2,8-linked Neu5Ac up to 400 residues long conferring important properties to the cells on which it is found [24]. In healthy adult tissues, the expression of polySia, referred to as polysialylation, is mainly limited to the cells of the nervous, immune, and reproductive systems, while many aggressive cancers display high levels of this unique glycan [24,25,26,27,28,29]. Increased polySia in cancer cells promotes resistance to apoptosis under hypoxic conditions, and immune evasion, cell migration, and invasiveness [30,31,32,33]. Moreover, aberrant sialylation is increasingly recognized as a hallmark of numerous challenging chronic diseases other than cancers, such as autoimmune diseases [34,35]. Of note, potential therapeutic effects of modulating sialyltransferase activity with different compounds were demonstrated both in vitro and in vivo [31,36,37,38]. As far as the implication of sialylation in skin fibrosis is concerned, a recent study reported that polySia is dysregulated in systemic sclerosis, a multisystem autoimmune disease characterized by fibrosis not only of the skin but also of various internal organs [39]. In particular, overexpression of polySia was mostly present in dermal fibroblasts of fibrotic skin, and both dermal and circulating polySia correlated with the degree of skin fibrosis, with the highest levels in systemic sclerosis patients with rapidly progressive diffuse skin fibrosis [39]. Nevertheless, whether aberrantly high levels of polySia or sialylation in general may be functionally implicated in fibroblast dysfunction and transition to myofibroblasts remains to be determined.

On these premises, we herein investigated whether inhibition of sialylation could interfere with the transition from skin fibroblasts to myofibroblasts induced by the master profibrotic mediator TGFβ1. To this aim, we employed the 3-Fax-peracetyl-Neu5Ac (3-Fax) compound that results in production of CMP-3-Fax-Neu5Ac, which is a competitive inhibitor of virtually all sialyltransferases and also causes negative feedback inhibition for the synthesis of CMP-Neu5Ac, thus resulting in the global blockade of sialylation [37,40,41].

## 2. Materials and Methods

### 2.1. In Vitro Culture of Human Skin Fibroblasts

Three primary cell lines of adult human skin fibroblasts were obtained, as previously reported [42]. Briefly, skin samples obtained as waste material from plastic surgery were subjected to a two-step immunomagnetic microbead-based cell separation that allowed the separation of three dermal cell types: CD31^−^/CD34^+^ telocytes, CD31^+^/CD34^+^ endothelial cells, and CD31^−^/CD34^−^ fibroblasts [42]. The CD31^−^/CD34^−^ fibroblasts were used in the present study. Cells were maintained at 37 °C and 5% CO_2_ under sterile conditions, and the culture medium, consisting of 4.5 g/L glucose Dulbecco’s modified eEagle medium (DMEM; 11-965-092; Thermo Fisher Scientific, Waltham, MA, USA) supplemented with 10% fetal bovine serum (FBS; ECS1104L; Euroclone, Milan, Italy), 1% L-glutamine (ECB3000D; Euroclone), and 1% penicillin/streptomycin (ECB3001D; Euroclone), was changed every 72 h. After reaching confluence, fibroblasts were pelleted or collected and subsequently seeded on different supports, depending on the specific experiment. Fibroblasts were used between the third and seventh passages in culture.

### 2.2. Cell Stimulation

Before each experiment, cells were starved for 2 h in basal medium containing 2% FBS, preincubated for an additional 2 h with 100 µM of the competitive pan-sialyltransferase inhibitor 3-Fax (566224; Sigma-Aldrich, St. Louis, MO, USA), and then challenged with 10 ng/mL recombinant human TGFβ1 (PeproTech, Rocky Hill, NJ, USA) for 48 h or 72 h to induce fibroblast-to-myofibroblast transition. Cells were also stimulated with TGFβ1 alone, without pretreatment with 3-Fax. The 3-Fax compound was dissolved in dimethyl sulfoxide to a final concentration of less than 0.1%. After 48 h stimulation, skin fibroblasts were examined for cell viability, proliferation, and mRNA expression, while protein expression and contractile capabilities were evaluated after 72 h. Confluence assessment was also performed after 48 h stimulation. Migration activity was tested after 24 h stimulation by in vitro scratch assay.

### 2.3. Annexin V/Propidium Iodide Flow Cytometer Assay

Human skin fibroblasts, seeded into 6-well plates until 90% confluence and stimulated for 48 h as described above, were harvested with Accutase (ECB3056D; Euroclone), transferred in flow cytometer tubes, and subsequently subjected to the annexin V/propidium iodide (PI) flow cytometer assay, as detailed elsewhere [43]. Samples were analyzed with a BD FACS Canto II flow cytometer (BD Biosciences, Franklin Lakes, NJ, USA), and the percentage of viable, early apoptotic, late apoptotic, and necrotic cells was calculated depending on annexin V and/or PI positivity. At least 10,000 events were collected for each sample, which were tested in triplicate. 

### 2.4. Cell Proliferation Assay

Cell proliferation, assessed on cells seeded onto 96-well plates (9 × 10^3^ cells per well) and stimulated for 48 h as previously described, was determined by means of the WST-1 assay (5015944001; Roche, Basilea, Switzerland), according to the instructions of the manufacturer. The experiment was performed with six technical replicates for each experimental condition, and data were expressed as the percentage of the increase or decrease in cell proliferation over the proliferative effect of 2% FBS-DMEM. 

### 2.5. Cell Morphology and Confluency Assessment

Cell morphology and confluency were both assessed under a Mateo TL RUO inverted microscope (Leica Microsystems, Mannheim, Germany). For cell confluency assessment, 5 × 10^5^ cells were seeded in T75 culture flasks. The percentage of the surface area covered by cells was evaluated with the Mateo TL RUO microscope confluency module after 48 h stimulation. Cell morphology was further determined by staining the F-actin cytoskeleton with Alexa 488-conjugated phalloidin (1:40 dilution; Invitrogen, Carlsbad, CA, USA) and counterstaining nuclei with 4′,6-diamidino-2-phenylindole (DAPI), followed by imaging with a Leica Stellaris 5 confocal laser scanning microscope equipped with the LAS X software (version 5.2.2; Leica Microsystems) using a Plan-Apo ×63/1.4NA oil immersion objective.

### 2.6. In Vitro Scratch Assay

Scratch assay was performed on confluent human skin fibroblasts seeded onto 6-well plates and cultured in complete DMEM. After 24 h of starvation, the medium was removed and the cell monolayer was scratched with a 200-μL pipette tip in order to obtain a ~1 mm wide area without cells. Once all detached cells were removed, the monolayers were incubated as described above. The cell migratory capacity was evaluated by capturing phase-contrast images of the scratched area under a Mateo TL RUO microscope (Leica Microsystems), with a ×4 objective. The pictures, acquired immediately after scratching and 24 h later, were compared to quantify the scratched area closure rate. For each cell line, all experimental conditions were performed in triplicate.

### 2.7. Quantitative PCR

Human skin fibroblast RNA content, purified after 48 h stimulation with the RNeasy Micro Kit (74004; Qiagen, Milan, Italy), was quantified by means of a NanoDrop 8000 Spectrophotometer (Thermo Fisher Scientific). RNA was then reverse transcribed to cDNA followed by gene expression assessment with SYBR Green real-time PCR, as previously described [44,45]. The list of oligonucleotide primer pairs (QuantiTect primer assays; Qiagen) is provided in Table 1. The 18S ribosomal RNA (Hs_RRN18S_1_SG; QT00199367; Qiagen) was used as the housekeeping gene for normalization. Differences in gene expression were calculated with the threshold cycle (Ct) and comparative Ct methodology for relative quantification. All experimental points were analyzed in triplicate for each of the three human skin fibroblast lines.

### 2.8. Western Blotting

After 72 h stimulation, cellular pellets were collected, and proteins were extracted by lysing cells in a solution containing Ripa buffer (89,901; Thermo Fisher Scientific), a complete protease inhibitor cocktail (11,697,498,001; Roche), 1 mM sodium orthovanadate, and 1 mM NaF. Once sonicated, proteins were quantified with the Bradford’s method and subsequently boiled at 90 °C for 5 min after the addition of Laemmli sample buffer (Bio-Rad, Hercules, CA, USA) and β-mercaptoethanol. Thirty µg of proteins was then loaded into a precast gel and, after the electrophoretic run, blotted onto a nitrocellulose membrane by using the Trans-Blot Turbo Mini 0.2 µm nitrocellulose transfer packs (#1704158; Bio-Rad) and the Trans-Blot Turbo transfer system instrument (Bio-Rad). The Western blotting analysis was performed according to previously published procedures [44,45], and the list of the primary antibodies used is shown in Table 2. Protein bands were detected with the ChemiDoc Touch Imaging System (Bio-Rad), and the densitometry of each band was calculated using ImageJ software 64-bit Java 1.8.0_172 Windows version (NIH, Bethesda, MD, USA; online at http://imagej.net/ij/, accessed on 15 January 2024).

### 2.9. Fluorescence Immunocytochemistry

To perform fluorescence immunocytochemistry, fibroblasts were seeded onto 20 × 20 mm glass coverslips, treated as previously described for 72 h, and finally fixed with 3.7% buffered paraformaldehyde. After permeabilizing cell membranes with 0.1% Triton X-100 in PBS for 10 min at room temperature, cells were washed with PBS, blocked with 1% bovine serum albumin in PBS for 1 h at room temperature, and subsequently incubated at 4 °C overnight with the following primary antibodies: rabbit monoclonal anti-polySia (1:100; RAB00125; Abnova, Taipei, Taiwan), mouse monoclonal anti-α-SMA (1:100; ab7817; Abcam, Cambridge, UK), and rabbit monoclonal anti-COL1A1 (1:300; #39952; Cell Signaling Technology, Danvers, MA, USA). Irrelevant isotype- and concentration-matched IgGs (Sigma-Aldrich) were used as negative controls. On the following day, the secondary antibodies Alexa Fluor-488-conjugated and Rhodamine Red-X-conjugated IgG (1:200; Invitrogen) were applied on coverslips for 45 min at room temperature in the dark, while nuclei were counterstained blue for 10 min at room temperature in the dark with DAPI. Coverslips were finally mounted onto glass slides and immunolabeled fibroblasts were observed and photographed with a Leica DM4000-B microscope furnished with a Leica DFC310 FX 1.4-megapixel digital color camera and the Leica software application suite LAS V3.8 (Leica Microsystems).

### 2.10. Collagen Gel Matrix Contraction Assay

The collagen gel matrix contraction assay was performed using a commercial kit (Floating Matrix Model; CBA-5020; Cell Biolabs, San Diego, CA, USA). Cells, treated for 72 h as described above, were firstly detached and resuspended in DMEM containing 2% FBS (2 × 10^6^ cells/mL). Then, 500 µL of a solution obtained by mixing 100 µL of cell suspension and 400 µL of collagen gel matrix solution were added to each well of an adhesion-resistant matrix coated 24-well plate. Cell-free gels were used as negative controls. After 1 h at 37 °C and 5% CO_2_, basal medium or medium containing different stimuli was added on top of each polymerized collagen gel matrix. The test was performed in triplicate for each experimental point. After 24 h, the plate was photographed and the area of each gel was measured with ImageJ software 64-bit Java 1.8.0_172 Windows version (NIH; online at http://imagej.net/ij/, accessed on 19 December 2023).

### 2.11. Statistical Analysis

Statistical analysis was performed with GraphPad Prism 5 software. After verification of data normality with the Kolmogorov–Smirnov test and further confirmation with the Shapiro–Wilk test, one-way analysis of variance (ANOVA) followed by post hoc Tukey’s test were carried out to compare three experimental groups. All data were expressed as mean ± standard deviation. Values of *p* < 0.05 were considered statistically significant.

## 3. Results

### 3.1. The Pan-Sialyltransferase Inhibitor 3-Fax Attenuates TGFβ1-Induced PolySia Expression in Human Skin Fibroblasts

**Figure 1:**
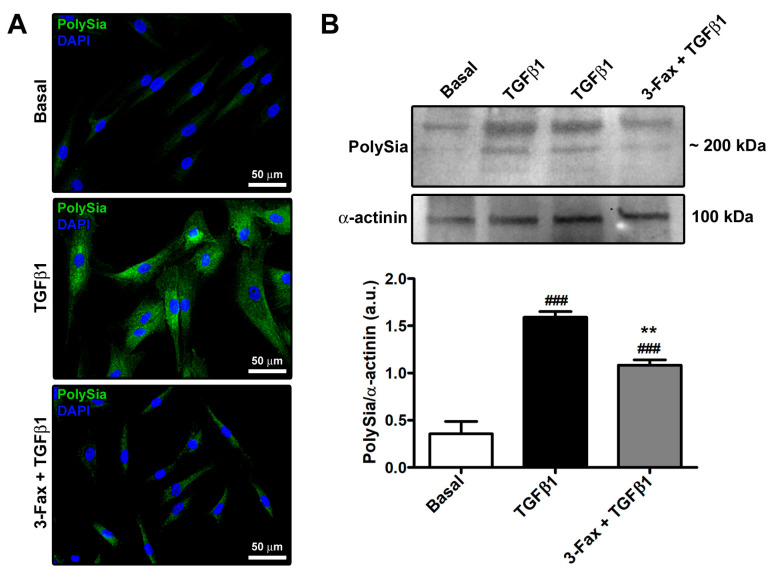
TGFβ1 stimulation of human skin fibroblasts induces a significant upregulation of polySia expression, an effect that is strongly reduced by inhibition of sialyltransferases with 3-Fax. (**A**) Representative fluorescence photomicrographs of skin fibroblasts immunostained for polySia and counterstained for nuclei with DAPI. Scale bar: 50 μm. (**B**) Demonstrative immunoblots for polySia, using α-actinin as a loading control for normalization. The two TGFβ1 points shown are technical replicates. Molecular weights (kDa) are shown. Optical density of the bands is expressed in arbitrary units (a.u.). Bars represent the mean ± standard deviation of three independent experiments (*n* = 3 technical replicates each) from three cell lines. ### *p* < 0.001 vs. basal, ** *p* < 0.01 vs. TGFβ1 (Tukey’s test). DAPI—4′,6-diamidino-2-phenylindole; 3-Fax—3-Fax-peracetyl-Neu5Ac; PolySia—polysialic acid; TGFβ1—transforming growth factor β1.

Preliminary immunofluorescence and Western blotting analyses of polySia content were performed on human skin fibroblasts at basal condition and stimulated with TGFβ1 alone or added 2 h after pre-incubation with the competitive pan-sialyltransferase inhibitor 3-Fax. As shown in Figure 1A,B, skin fibroblasts at basal condition showed negligible expression of polySia. Stimulation with profibrotic TGFβ1 had the capability to induce polySia expression, and such an effect could be significantly attenuated by pretreating skin fibroblasts with 3-Fax (Figure 1A,B).

### 3.2. The Pan-Sialyltransferase Inhibitor 3-Fax Inhibits TGFβ1-Induced Proliferation of Human Skin Fibroblasts

**Figure 2:**
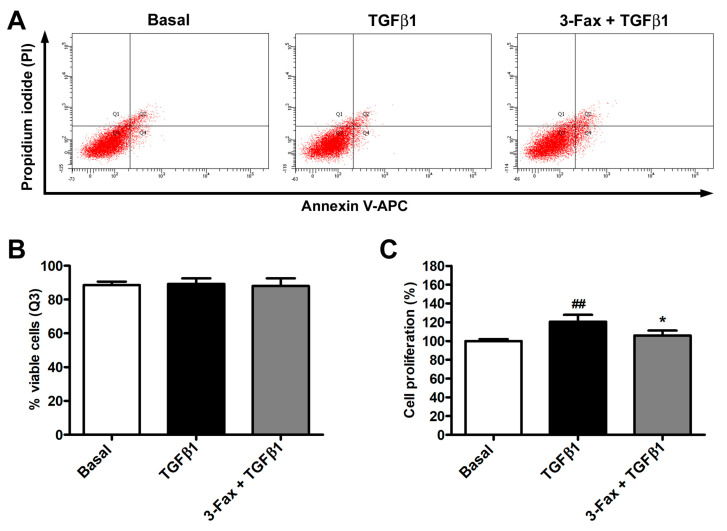
The pan-sialyltransferase inhibitor 3-Fax does not affect the viability of human skin fibroblasts and reduces TGFβ1-induced cell proliferation. (**A**) Demonstrative annexin V/PI flow cytometry assay plots of skin fibroblasts at basal condition and challenged with TGFβ1 alone or added 2 h after pre-incubation with 3-Fax. Q1 quadrant shows annexin V^−^/PI^+^ necrotic cells, Q2 quadrant annexin V^+^/PI^+^ late apoptotic cells, Q3 quadrant annexin V^−^/PI^−^ viable cells, and Q4 quadrant annexin V^+^/PI^−^ early apoptotic cells. (**B**) Percentage of viable cells (Q3 quadrant) for each experimental point. (**C**) Cell proliferation evaluated with WST-1 colorimetric assay. The proliferative rate at basal condition is set to 100%, and the other results are normalized consequently. Bars represent the mean ± standard deviation of three independent experiments (*n* = 3 technical replicates each for cell viability assay, *n* = 6 technical replicates each for WST-1 assay) from three cell lines. ## *p* < 0.01 vs. basal, * *p* < 0.05 vs. TGFβ1 (Tukey’s test). 3-Fax—3-Fax-peracetyl-Neu5Ac; PI—propidium iodide; TGFβ1—transforming growth factor β1.

In order to exclude any potential side effect of the pan-sialyltransferase inhibitor 3-Fax, we evaluated human skin fibroblast viability by using the annexin V/PI flow cytometry assay. As proven by the absence of significant differences in the number of viable, early apoptotic, late apoptotic, and necrotic cells amongst the different experimental points, cell viability was not affected neither by TGFβ1 alone or in combination with 3-Fax (Figure 2A,B). Regarding cell proliferation, evaluated by WST-1 assay, stimulation with TGFβ1 alone determined a significant, though not impressive, increase in the fibroblast proliferative rate, as reported in previous studies [45,46,47], while pretreatment with 3-Fax was able to reduce TGFβ1-induced skin fibroblast proliferation (Figure 2C).

### 3.3. Cell Confluency and Cell Migratory Capability Determination

**Figure 3:**
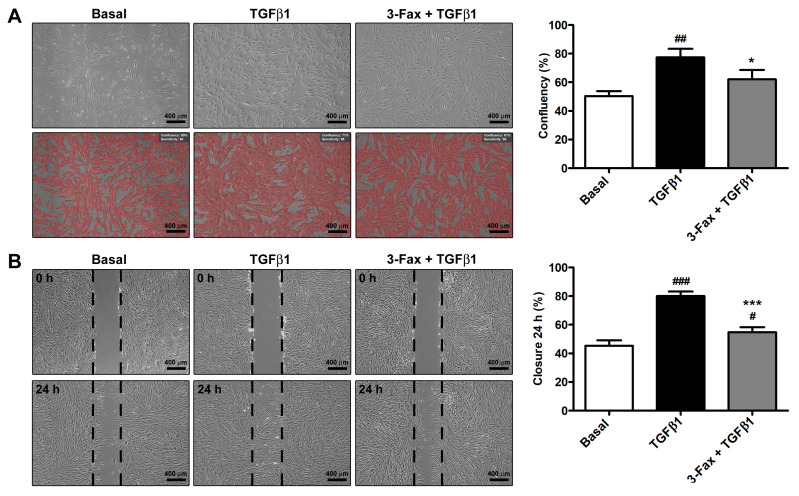
The pan-sialyltransferase inhibitor 3-Fax significantly lessens TGFβ1-induced capability of human skin fibroblasts to proliferate and regenerate the integrity of the cellular monolayer after scratching. Cell confluency and migration are assessed on skin fibroblasts cultured at basal condition and challenged with TGFβ1 alone or added 2 h after preincubation with 3-Fax. (**A**) Representative phase-contrast photomicrographs of the fibroblast monolayer and quantification of cell confluency after 48 h stimulation. (**B**) Demonstrative phase-contrast images of the scratched area at 0 and 24 h. Scale bar: 400 μm. The borders of the scratched area are drawn in black. Bars represent the mean ± standard deviation of three independent experiments (*n* = 3 technical replicates each) from three cell lines. ### *p* < 0.001, ## *p* < 0.01, and # *p* < 0.05 vs. basal, *** *p* < 0.001 and * *p* < 0.05 vs. TGFβ1 (Tukey’s test). 3-Fax—3-Fax-peracetyl-Neu5Ac; TGFβ1—transforming growth factor β1.

The ability of TGFβ1 to foster cell proliferation was confirmed by the high confluency percentage of TGFβ1-treated fibroblasts (Figure 3A). On the contrary, skin fibroblasts pre-incubated with 3-Fax before being challenged with TGFβ1 showed a significantly reduced confluency percentage, demonstrating the ability of 3-Fax to inhibit TGFβ1-induced proliferative effect (Figure 3A).

Stimulation of human skin fibroblasts with TGFβ1 significantly augmented their migratory ability, with ~85% scratched area closure after 24 h (Figure 3B). Pre-administration of 3-Fax resulted in a reduction of the TGFβ1-mediated effect, significantly lowering the scratched area closure percentage up to ~60% (Figure 3B).

### 3.4. Cell Morphology and Myofibroblast-Like Phenotype Assessment 

**Figure 4:**
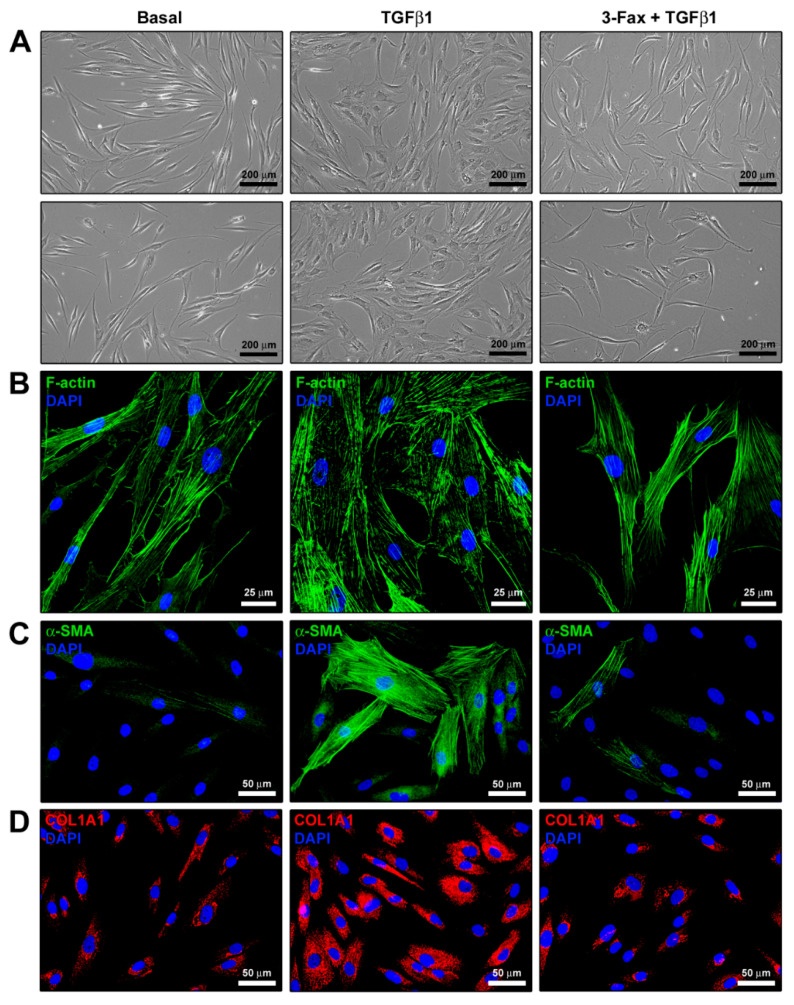
The pan-sialyltransferase inhibitor 3-Fax significantly inhibits the TGFβ1-induced acquisition of a profibrotic myofibroblast-like phenotype of human skin fibroblasts. (**A**) Demonstrative phase-contrast images of cells cultured at basal condition and challenged with TGFβ1 alone or added 2 h after preincubation with 3-Fax. (**B**) Representative fluorescence photomicrographs of skin fibroblasts stained for F-actin with Alexa 488-conjugated phalloidin. Nuclei are counterstained with DAPI. (**C**,**D**) Illustrative fluorescence images of skin fibroblasts immunostained for α-SMA and COL1A1 and counterstained for nuclei with DAPI. Scale bars: 200 μm (**A**), 25 μm (**B**), 50 μm (**C**,**D**). α-SMA—α-smooth muscle actin; COL1A1—α-1 chain of type I collagen; DAPI—4′,6-diamidino-2-phenylindole; F-actin—filamentous actin; 3-Fax—3-Fax-peracetyl-Neu5Ac; TGFβ1—transforming growth factor β1.

TGFβ1 treatment induced significant morphological changes in human skin fibroblasts, making them acquire a myofibroblast-like morphology characterized by a larger size and a flattened and polygonal-shaped body (Figure 4A,B). In addition, upon stimulation with TGFβ1, cells went through a substantial reorganization of the F-actin cytoskeleton, showing more abundant stress fibers (Figure 4A,B). Of note, pretreatment with 3-Fax was able to reduce all the TGFβ1-induced morphological and cytoskeletal changes (Figure 4A,B). As far as the assessment of myofibroblast-like features was concerned, fluorescence immunocytochemistry revealed that TGFβ1 treatment induced a significant upregulation of both intracellular α-SMA and COL1A1, with α-SMA being highly assembled into stress fibers (Figure 4C,D). Such an effect was strongly attenuated by the pre-administration of the pan-sialyltransferase inhibitor 3-Fax (Figure 4C,D). 

### 3.5. TGFβ1-Induced Acquisition of Myofibroblast Markers and Contractile Ability by Human Skin Fibroblasts Is Reduced by Preadministration of 3-Fax

**Figure 5:**
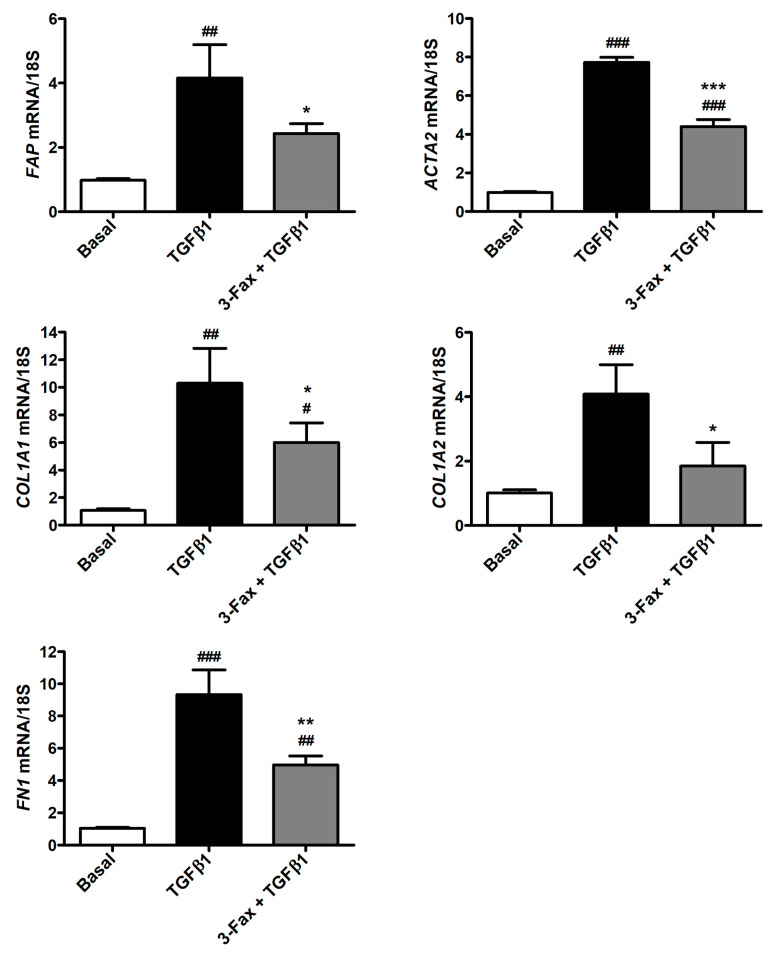
The pan-sialyltransferase inhibitor 3-Fax significantly reduces TGFβ1-induced expression of genes encoding myofibroblast markers in human skin fibroblasts. The expression of *FAP* (fibroblast activation protein), *ACTA2* (α-smooth muscle actin), *COL1A1* (α-1 chain of type I collagen), *COL1A2* (α-2 chain of type I collagen), and *FN1* (fibronectin 1) genes is quantified by quantitative real-time PCR. Basal expression is set to 1 for each gene and the other results are normalized accordingly. 18S ribosomal RNA is employed as housekeeping gene. Histograms represent the mean ± standard deviation of three independent experiments (*n* = 3 technical replicates each) from three cell lines. ### *p* < 0.001, ## *p* < 0.01, and # *p* < 0.05 vs. basal, *** *p* < 0.001, ** *p* < 0.01, and * *p* < 0.05 vs. TGFβ1 (Tukey’s test). 3-Fax—3-Fax-peracetyl-Neu5Ac; TGFβ1—transforming growth factor β1.

Quantitative PCR executed on human skin fibroblasts stimulated with TGFβ1 alone indicated a significant increase in the expression of *FAP*, *ACTA2*, *COL1A1*, *COL1A2*, and *FN1* genes (Figure 5). Of note, the pre-administration of 3-Fax to cells was effective to significantly lower the mRNA levels of all the aforementioned genes (Figure 5). 

**Figure 6:**
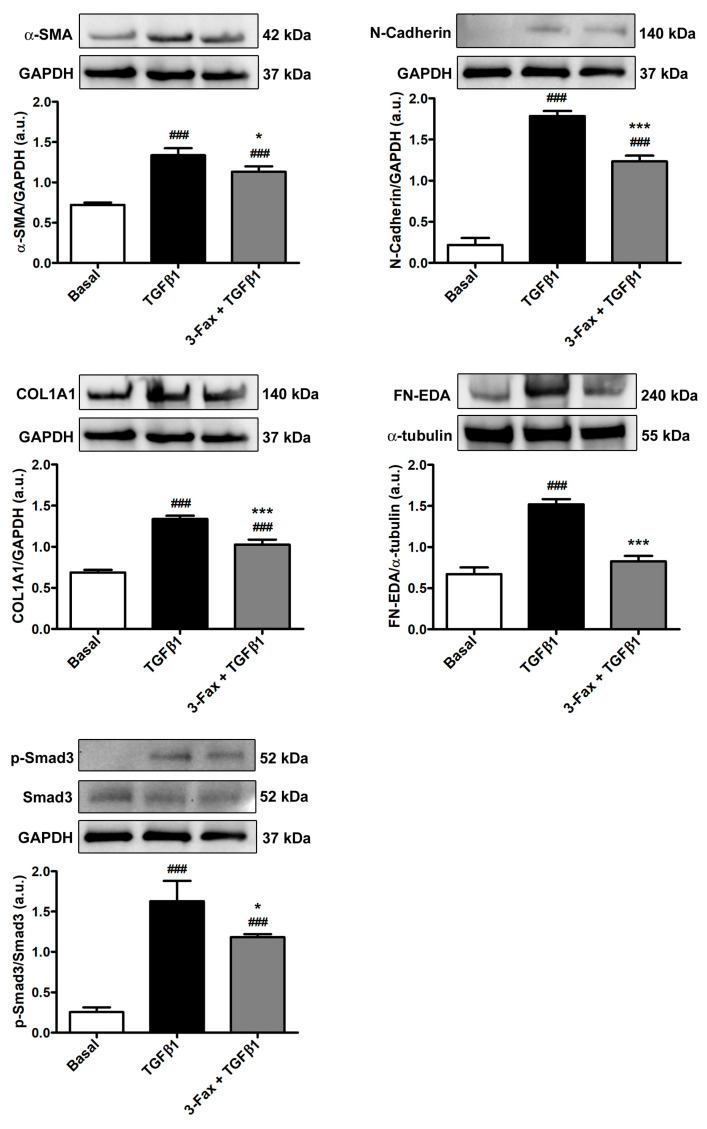
The pan-sialyltransferase inhibitor 3-Fax significantly decreases TGFβ1-induced protein expression of myofibroblast markers and Smad3-dependent canonical TGFβ1 signaling in human skin fibroblasts. Representative immunoblots for α-SMA, N-cadherin, COL1A1, FN-EDA, p-Smad3, and total Smad3. GAPDH and α-tubulin are measured as loading controls for normalization. The molecular weight (kDa) of each protein is shown. Optical density of the bands is expressed in arbitrary units (a.u.). Bars represent the mean ± standard deviation of three independent experiments (*n* = 3 technical replicates each) from three cell lines. ### *p* < 0.001 vs. basal, *** *p* < 0.001 and * *p* < 0.05 vs. TGFβ1 (Tukey’s test). α-SMA—α-smooth muscle actin; COL1A1—α-1 chain of type I collagen; 3-Fax—3-Fax-peracetyl-Neu5Ac; FN-EDA—fibronectin containing the alternatively spliced extra domain A; GAPDH—glyceraldehyde 3-phosphate dehydrogenase; p-Smad3—phosphorylated-Smad3; TGFβ1—transforming growth factor β1.

These findings were subsequently confirmed by Western blotting analysis, which indeed showed a significant increase in the expression not only of α-SMA and COL1A1 but also of myofibroblast-associated N-cadherin and fibronectin containing the alternatively spliced extra domain A, commonly referred to as FN-EDA, in TGFβ1-treated skin fibroblasts (Figure 6). The TGFβ1-induced upregulation of all these protein markers was significantly inhibited in skin fibroblasts pretreated with 3-Fax (Figure 6). 

Since Smad3 phosphorylation represents an important step in the profibrotic canonical TGFβ1 pathway, we also evaluated the protein levels of phosphorylated-Smad3/total Smad3 in each experimental condition. As shown in Figure 6, skin fibroblast stimulation with TGFβ1 alone strongly increased Smad3 phosphorylation, an effect that was significantly lessened by pretreatment with 3-Fax.

**Figure 7:**
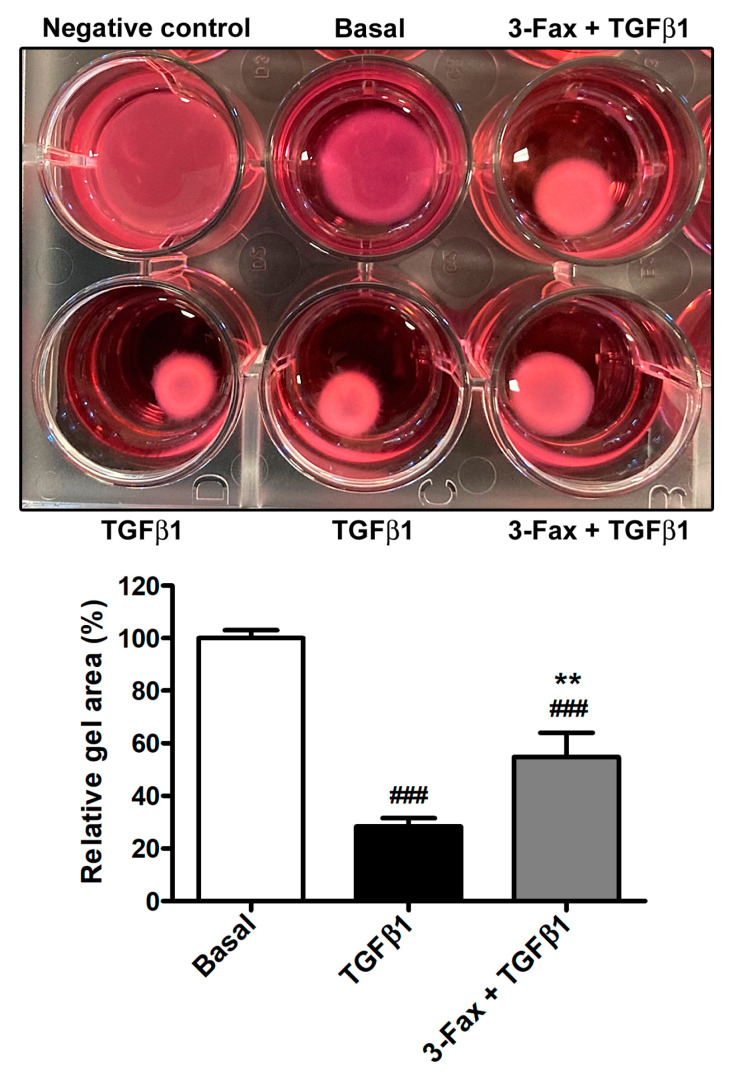
The pan-sialyltransferase inhibitor 3-Fax significantly dampens TGFβ1-induced myofibroblast-like contractile ability of human skin fibroblasts. Representative wells of the collagen gel contraction assay plate are shown in the upper panel. Gel area is expressed as the percentage of that detected for cells at basal condition. Bars represent the mean ± standard deviation of three independent experiments (*n* = 3 technical replicates each) from three cell lines. ### *p* < 0.001 vs. basal, ** *p* < 0.01 vs. TGFβ1 (Tukey’s test). 3-Fax—3-Fax-peracetyl-Neu5Ac; TGFβ1—transforming growth factor β1.

Finally, the pre-administration of 3-Fax to skin fibroblasts also led to a significant decrease in the TGFβ1-induced myofibroblast-like cell capability to contract collagen gel matrices (Figure 7).

## 4. Discussion

Fibrotic skin conditions such as pathological scarring and systemic sclerosis mainly manifest with fibroblast proliferation and differentiation into a profibrotic myofibroblast phenotype, causing an exaggerated and prolonged wound healing response that leads to dermal ECM hyperplasia [1,2,11,13,15,16,18]. Currently, the pathogenesis of these diseases has not been fully elucidated, and remarkably high medical needs and poor treatment possibilities still represent major burdens [1,12]. Therefore, an in-depth understanding of the mechanisms that regulate myofibroblast differentiation and, hence, potentially represent novel therapeutic targets is crucial to prevent the progression of the cutaneous fibrogenic process, or even revert established skin fibrosis [4,11,18]. In this regard, although increased levels of polySia were recently found in dermal fibroblasts of systemic sclerosis patients and correlated with the severity of skin fibrosis, any pathogenic implication of polySia or sialylation in general via possible effects on fibroblasts remains unknown [39]. By taking advantage of the in vitro model of adult human skin fibroblast-to-myofibroblast transition induced by recombinant human TGFβ1, our results demonstrate that an increase in polySia is a functional piece in the profibrotic differentiation of skin fibroblasts. Indeed, pretreatment of skin fibroblasts with the competitive global sialyltransferase inhibitor 3-Fax significantly dampened both the TGFβ1-induced raise in polySia levels and TGFβ1-induced proliferation, migration, changes in cell morphology, and phenotypic and functional differentiation into myofibroblasts, as testified by a significant reduction in *FAP*, *ACTA2*, *COL1A1*, *COL1A2*, and *FN1* gene expression; α-SMA, N-cadherin, COL1A1, and FN-EDA protein levels; and reduced stress fiber formation and contractile capability. Since activation of the Smad3-dependent pathway by TGFβ1 stimulation is known to be required for myofibroblastic differentiation of quiescent fibroblasts [18,48], we further examined whether the reduction of sialylation with the pan-sialyltransferase inhibitor 3-Fax had an effect on Smad3 phosphorylation. Western blot analysis disclosed that activation of the Smad3-dependent canonical TGFβ1 signaling was significantly reduced in skin fibroblasts pre-administered with 3-Fax. However, we cannot exclude that the blockade of sialylation could also interfere with non-canonical (non-Smad) TGFβ pathways in skin fibroblasts [18], which deserves further investigation. Moreover, whether canonical or non-canonical TGFβ signaling might regulate sialyltransferases in fibroblasts will merit in-depth analyses from a mechanistic point of view. In addition, it is important to consider that sialylation of the integrin ligand vitronectin was found to regulate stress fiber formation and cell spreading of dermal fibroblasts via a heparin-binding site [49], and that reciprocal TGFβ–integrin signaling is deeply implicated in ECM remodeling and fibrosis [50]. For instance, it was demonstrated that TGFβ receptor signaling can modulate β1- and β4-integrin sialylation [51,52]. Therefore, future research should dissect the putative contribution of sialylation in the fibroblast-to-myofibroblast transition via the modulation of the integrin-TGFβ crosstalk.

Of note, our results are consistent with a previous report showing that TGFβ1-induced expression of α-SMA in skin fibroblasts during the process to senescence is inhibited by the depletion of sialic acids with GalNAc-α-*O*-benzyl or sialidase treatment [53]. Furthermore, it is interesting to underline that profibrotic myofibroblasts are regarded as cancer-like cells in terms of increased invasiveness, resistance to apoptosis, proliferation, and genomic instability [4,18,39]. Hence, in agreement with our findings on fibroblasts differentiated into myofibroblasts via TGFβ1 treatment, both the proliferation and migration capacity of tumor cells were significantly inhibited by administration of the 3-Fax compound [54]. In addition, a recent study employing 3-Fax has shown increased sialylation in cancer-associated fibroblasts, which express α-SMA and have a myofibroblast-like phenotype [55]. Notably, it was demonstrated that cancer-associated fibroblasts can drive the differentiation of monocytes to immunosuppressive tumor-associated macrophages in vitro via sialic acid interactions with Siglecs, which are expressed on innate and adaptive immune cells [55]. Considering the importance of fibroblast-immune cell interactions in the initiation and progression of the fibrotic process [2,11,16,18,20,21], the occurrence of similar mechanisms should be explored in the context of skin fibrosis.

These results are important as they suggest that sialylation is worthy of further exploration as a possible target for therapies against fibrosing skin disorders. We restricted our investigation to primary human skin fibroblasts as the first in vitro model essential for testing our working hypothesis because of their ease of acquisition, culture, and transition toward profibrotic myofibroblasts. Since in the present study we used a preventive approach (i.e., the blockade of sialylation prior to the induction of fibroblast-to-myofibroblast transition by TGFβ1), it would be interesting to analyze whether the blocking of sialylation might even reverse or stop myofibroblast differentiation and matrix synthesis in a curative setting in vitro. Whether the inhibition of sialylation would show the same antifibrotic ability in preclinical in vivo models of skin fibrosis remains to be determined. For example, we foresee that this could be achieved using the well-established mouse model induced by repeated subcutaneous injections of bleomycin, which features skin immune reactions and inflammation followed by development of fibrosis [20,56,57,58]. Even considering the important biological roles played by polySia and other sialosides in the regulation of the immune response [28,35,59], such an experimental system will allow to dissect the role of sialylation in cell signaling interactions of skin fibroblasts with the surrounding microenvironment bearing inflammatory/immune cells that release TGFβ1 along with multiple other profibrotic stimuli. Although in our in vitro study we employed the 3-Fax sialic acid analog that inhibits virtually all sialyltransferases [37,41], we should consider that the use of this compound in vivo may have some limitations, at least if systemically administered, because of deleterious effects on liver and kidney function [40]. Therefore, exploiting more selective sialyltransferase inhibitors such as 8-keto-Neu5Ac, which was found to be nontoxic at effective concentrations and to block polySia synthesis in cancer cell lines with minimal effects on other sialyl glycoforms [37,60], is advisable for future in vivo experimentation. Finally, we acknowledge that, following the herein demonstration of the ability of sialylation blockade to prevent fibroblast-to-myofibroblast transition, further work is necessary to demonstrate whether the same approach might also be effective in inducing myofibroblast dedifferentiation and skin fibrosis regression.

As far as the experimental design of our study is concerned, it is necessary to point out that we did not incorporate a treatment with the sialyltransferase inhibitor 3-Fax alone based on the following rationale. First, since we used the in vitro model of TGFβ1-induced fibroblast-to-myofibroblast transition to test our hypothesis that polySia expression could be induced by TGFβ1 stimulation and have a functional implication in the transition process, the basal condition (i.e., untreated cells) was considered the experimental control. This was essential to verify that the in vitro model was working properly (i.e., stimulation with recombinant human TGFβ1 effectively induced the fibroblast-to-myofibroblast transition). Moreover, as known from much of the literature [24,25,26,27,28,29], we considered that the expression of polySia is restricted to a few anatomical districts and cell types in healthy conditions. Indeed, we found negligible expression of polySia in skin fibroblasts at basal condition (Figure 1A,B). In addition, sialyltransferase inhibitors are compounds that specifically block sialylation with no other expected cellular effects [36]. Of note, similarly to our experimental design, the study of Sasaki et al. [53] did not include a treatment with the sialyltransferase inhibitor alone for experiments on fibroblasts treated with TGFβ1. Nevertheless, we considered essential an inclusion of cell viability assessment in our experimental design in order to exclude that results of gene and protein expression levels of the myofibroblast markers could be biased by an increase in cell death in the presence of the sialyltransferase inhibitor. As clearly shown in Figure 2A,B, we verified that the sialyltransferase inhibitor did not alter the viability of skin fibroblasts.

## 5. Conclusions

In summary, the present in vitro study demonstrates for the first time that (i) aberrant polysialylation occurs during profibrotic activation of adult human skin fibroblasts by TGFβ1 and (ii) blockade of sialylation with competitive inhibition of sialyltransferases can effectively interfere with the TGFβ1-driven fibroblast-to-myofibroblast transition process. In perspective, we are confident that these findings provide the necessary groundwork for further preclinical in vitro and in vivo studies to untangle how sialylation could contribute to making the difference between normal wound healing and pathological scarring, and whether its modulation could afford new antifibrotic therapeutic opportunities.

## Figures and Tables

**Table 1:** Oligonucleotide primer pairs used for quantitative PCR.

Gene	Assay ID	Catalog Number
*FAP*	Hs_FAP_1_SG	QT00074963
*ACTA2*	Hs_ACTA2_1_SG	QT00088102
*COL1A1*	Hs_COL1A1_1_SG	QT00037793
*COL1A2*	Hs_COL1A2_1_SG	QT00072058
*FN1*	Hs_FN1_1_SG	QT00038024

**Table 2:** Primary antibodies used for Western blotting.

Primary Antibody	Host Species	Catalog Number	Producer	Dilution
anti-polySia	rabbit	RAB00125	Abnova	1:1000
anti-α-SMA	mouse	ab7817	Abcam	1:300
anti-N-cadherin	rabbit	#13116S	Cell Signaling Technology	1:1000
anti-COL1A1	rabbit	#39952	Cell Signaling Technology	1:1000
anti-fibronectin	mouse	SAB4200880	Sigma-Aldrich	1:1000
anti-p-Smad3	rabbit	#9520S	Cell Signaling Technology	1:1000
anti-α-actinin	rabbit	#3134	Cell Signaling Technology	1:1000
anti-GAPDH	mouse	ab8245	Abcam	1:5000
anti-α-tubulin	rabbit	#2144	Cell Signaling Technology	1:1000

α-SMA—α-smooth muscle actin; polySia—polysialic acid; p-Smad3—phosphorylated-Smad3; GAPDH—glyceraldehyde 3-phosphate dehydrogenase.

## Data Availability

The original contributions presented in the study are included in the article, further inquiries can be directed to the corresponding author.
